# Outcomes of Early versus Late Endotracheal Intubation in Patients with Initial Non-Shockable Rhythm Cardiopulmonary Arrest in the Emergency Department

**DOI:** 10.1155/2021/2112629

**Published:** 2021-12-28

**Authors:** Kiattichai Daorattanachai, Winchana Srivilaithon, Vitchapon Phakawan, Intanon Imsuwan

**Affiliations:** Department of Emergency Medicine, Faculty of Medicine, Thammasat University, Pathum Thani 12120, Thailand

## Abstract

**Background:**

Sudden cardiac arrest is a critical condition in the emergency department (ED). Currently, there is no considerable evidence supporting the best time to complete advanced airway management (AAM) with endotracheal intubation in cardiac arrest patients presented with initial non-shockable cardiac rhythm.

**Objectives:**

To compare survival to hospital discharge and discharge with favorable neurological outcome between the ED cardiac arrest patients who have received AAM with endotracheal intubation within 2 minutes (early AAM group) and those over 2 minutes (late AAM group) after the start of chest compression in ED.

**Methods:**

We conducted a retrospective cohort study involving the ED cardiac arrest patients who presented with initial non-shockable rhythm in ED. Multivariable logistic regression analysis was used to evaluate the independent effect of early AAM on outcomes. The outcomes included the survival to hospital discharge and discharge with favorable neurological outcome.

**Results:**

There were 416 eligible participants: 209 in the early AAM group and 207 participants in the late AAM group. The early AAM group showed higher survival to hospital discharge compared with the late AAM group, but no statistically significant difference (adjusted odds ratio (aOR): 1.28, 95% confidence interval (CI): 0.59 -2.76, *p* = 0.524). Discharge with favorable neurological outcome is also higher in the early AAM group (aOR: 1.68, 95% CI, 0.52 -5.45, *p* = 0.387).

**Conclusion:**

This study did not demonstrate a significant improvement of survival to hospital discharge and discharge with favorable neurological outcome in the ED cardiac arrest patients with initial non-shockable cardiac arrest who underwent early AAM within two minutes. More research is needed on the timing of AAM and on airway management strategies to improve survival.

## 1. Introduction

Cardiac arrest is a critical condition seen in emergency departments worldwide with incidence rates of 330,000 per year in the US and 275,000 in the European Union [1].

High-quality cardiopulmonary resuscitation (CPR), including effective chest compression, minimized interruption of compression, and appropriate assisted ventilation, is a crucial step in the survival of patients with cardiac arrest. Hypoxia is the most frequent cause of in-hospital cardiac arrest from a non-cardiac cause, approximately 20% of all causes [2, 3]. Thus, ventilatory strategy is important during CPR, especially in initial non-shockable are mostly caused by non-cardiac causes.

Bag-mask ventilation (BMV) with or without basic airway adjunct is commonly used as an initially assisted ventilation in cardiac arrest patients. Some patients need advanced airway management (AAM) with endotracheal intubation or insertion of a supraglottic airway device during CPR. The optimal method of ventilation during cardiac arrest remains controversial.According to the 2020 American Heart Association (AHA) guidelines for cardiopulmonary resuscitation and emergency cardiovascular care, BMV or an AAM may be considered during CPR in any setting [4].

Endotracheal intubation provides many advantages such as improved oxygenation, expel carbon dioxide, improved chest compression fraction, and measurement of end-tidal carbon dioxide for monitoring the quality of chest compression [5]. However, this procedure might increase the risk of interruption of chest compression, esophageal intubation, or undesirable hyperventilation [4].

The ED of Thammasat University Hospital uses advanced airway strategy with endotracheal intubation for resuscitation of cardiac arrests. Initial BMV in the first cycle of basic-life-support was followed by endotracheal intubation. Supraglottic airway devices, however, were only used in the failed intubation.

The previous study showed very high tracheal intubation success rates with 96.7% success within three attempts [6]. The present AHA guidelines recommend that, in the setting of high tracheal intubation success rates, advanced airway strategy can be used. However, no evidence currently exists demonstrating the optimal time to AAM. Wong et al. [7] revealed that AAM less than 5 minutes improves survival at discharge. Bobrow et al. showed the benefit of delayed AAM in the prehospital setting [8]. This study evaluated the effect of early AAM compared with late AAM in cardiac arrest patients presented with initial non-shockable cardiac rhythm.

## 2. Methods

This was a retrospective cohort study that was conducted in the emergency department of Thammasat University Hospital (TUH), Pathum Thani, Thailand, between January 2013 and October 2019. TUH is an 800-bed tertiary academic teaching hospital in the suburbs north of Bangkok, with approximately 1.1 million people living in the area with 60, 000 patients visiting ED annually.

### 2.1. Participants

Eligible patients included all emergency department cardiac arrest (EDCA) patients over 18 years and presenting with initially non-shockable cardiac rhythm (asystole or pulseless electrical activity) who had an advanced airway placement during CPR. We excluded patients with do not attempt resuscitation (DNAR) orders, intubation prior to cardiac arrest, out-of-hospital intubation, and referral of the patient to another hospital after resuscitation.

### 2.2. Variables

We recorded data on a standard form, which included gender, age, initial location of cardiac arrest, etiology of cardiac arrest, initial cardiac rhythm, witnessed cardiac arrest, bystander CPR, collapsed time to first chest compression, time to first dose of epinephrine, time to AAM, defibrillation during resuscitation, and intravenous medications during resuscitation. AAM referred to endotracheal intubation, surgical cricothyroidotomy, and tracheostomy. Favorable neurological outcome was defined by cerebral performance category (CPC) 1 or 2. EDCA includes 3 groups of patients. (1) Out-of-hospital cardiac arrests who received prehospital CPR but without recovery and need to continue CPR when arrived at ED, (2) out-of-hospital cardiac arrests who did not receive prehospital CPR when arrived at ED, and (3) in-hospital cardiac arrests who received first chest compression in ED. Time to advanced airway management is the time from the first chest compression at ED to the successful placement of the advanced airway. Early AAM and late AAM were defined as the completion of advanced airway placement within two minutes and over two minutes, respectively.

### 2.3. Data Source/Measurement

Data were collected from Thammasat CPR Registry and the patient's medical charts. The outcomes were the survival to hospital discharge or within 30 days and the survival with favorable neurological outcome.

### 2.4. Study Size

From our pilot study in the emergency department, Thammasat University Hospital, survival to hospital discharge in early AAM and late AAM was 14% and 6%, respectively. STATA software was used to calculate the sample size. To achieve 80% power to detect the difference, a sample size of 198 patients in each group was estimated for a 1-sided test and an alpha level of 5%.

### 2.5. Statistical Methods

Quantitative variables were presented as mean and standard variations. Categorial variables were expressed as percentages and compared using the chi-squared test. Multivariable logistic regression analysis was used to evaluate the independent effect of early AAM on survival outcomes. Statistical significance was defined as a *p* value <0.05. All analyses were performed using STATA software (version 14.0, StataCorp, College Station).

### 2.6. Ethics Approval

The study was approved by the Human Research Ethics Committee of Thammasat University, Faculty of Medicine (MTU-EC-EM-0-209/61).

## 3. Results

### 3.1. Participants

**Figure 1:**
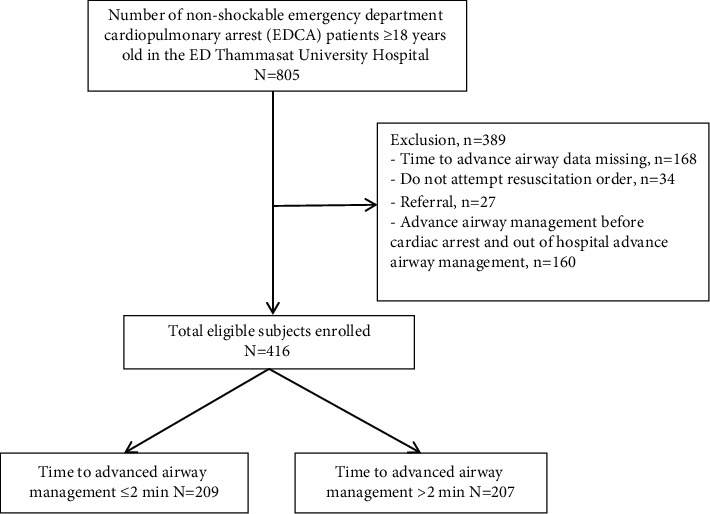
Flow diagram of the study population enrolled.

From January 2013 to October 2019, 805 patients with non-shockable cardiac arrest were identified. Four hundred and sixteen patients were eligible for the study, 209 patients (50.24%) received AAM within two minutes, and 207 patients (49.76%) received that over two minutes. 389 patients were excluded due to data missing (168), DNAR order (34), referral (27), and AAM before cardiac arrest or out-of-hospital advanced airway (160) (see Figure 1).

### 3.2. Descriptive Data

Clinical characteristics and demographic data are summarized in Table 1. The mean time of AAM in the early AAM group was 1.16 minutes (SD 0.83) compared with 5.87 minutes (SD 5.35) in the late AAM group.

The early AAM group received epinephrine earlier than the late AAM group (1.39 versus 2.28 minutes, *p* < 0.001). There was no evidence of a statistical difference in gender, age, initial location of cardiac arrest, etiology of cardiac arrest, witnessed cardiac arrest, bystander CPR, collapsed time to first chest compression, initial rhythm of cardiac arrest, defibrillation during resuscitation, intravenous amiodarone used during resuscitation, and intravenous calcium administration.

### 3.3. Main Results

Return of spontaneous circulation (ROSC) occurred in 106 patients (50.72%) in the early AAM group and 98 patients (47.34%) in the late AAM group (*p* = 0.094). Survival to hospital discharge was 23 patients (11%) in the early AAM group and 14 patients (6.80%) in the late AAM group (*p* = 0.168). Discharge with favorable neurological outcome was 13 patients (6.25%) and 6 patients (2.91%) in the early AAM and the late AAM group, respectively (*p* = 0.157) (see Table 2).

Multivariable logistic regression analysis was used to control the following variables: time to first dose epinephrine administration, sodium bicarbonate use, age, gender, etiology of cardiac arrest, and collapsed time to first chest compression. The early AAM group showed higher survival to hospital discharge compared with the late AAM group, but without statistically significant difference (adjusted odds ratio (aOR): 1.28, 95% confidence interval (CI); 0.59–2.76, *p*=0.524) (see Table 3).

The early AAM group also had a higher favorable neurological outcome but without statistically significant difference (aOR: 1.68, 95%CI; 0.52–5.45, *p* = 0.387) (see Table 4).

## 4. Discussion

Early advanced airway management within two minutes after the first chest compression at ED in emergency department cardiac arrest patients presented with non-shockable cardiac rhythm showed higher survival to hospital discharge and survival with favorable neurological outcome compared with late over two minutes; however, there was no evidence of a significant difference between the study groups.

Although this study showed that a presumed cardiac etiology was the common cause of cardiac arrest (56%), it was lower than the study from Izawa et al. (73.9%) [9]. However, approximately 19% of all cardiac arrests were due to hypoxia, which is comparable with other studies [2, 3]. The mean time of airway insertion was 1.6 minutes in the early AAM group while Wong et al. reported the mean time of 2.1 minutes in a group who were intubated before 5 minutes [7].

Even though the witnessed cardiac arrest was 71.11% (296/416), 10.6% (44/416) of whom received bystander CPR. To date, the knowledge and skill in basic life support in the general population is generally low. The EMS system in Thailand is underdevelopment. The bystander CPR rate in our country is lower than the developed country. Therefore, time to first chest compression is longer than the developed country.

About half of our patients (49%) had a return of spontaneous circulation, essentially the same as that reported by Wong et al. (50.3%) [7]. Our overall survival to hospital discharge was 8% compared with 13.5% reported by Wang et al. [10].

Only 4.5% of the patients had discharge with favorable neurological outcome, whereas Wong et al. [7] had a better outcome at 11%. We assume that the poorer outcome in our study could be explained by a non-cardiac cause of cardiac arrest (44%) compared with another study (26.1%) [9], which is usually associated with a poor prognosis.

The delayed administration of epinephrine is associated with a lower rate of survival in cardiac arrest with non-shockable rhythm [11]. The 2019 AHA Focused Update on Advanced Cardiovascular Life Support [12] emphasized the early epinephrine administration in non-shockable cardiac arrest. From our study, early epinephrine administration was associated with early AAM. Izawa et al. [9] demonstrated that in their early AAM group, their patients also received early epinephrine administration. In contrast, Lupton et al. [13] did not find an association between AAM and time to initial epinephrine administration in out-of-hospital cardiac arrest. This finding might be explained by the effect of high performance of the CPR team; with more effective resuscitation, epinephrine can be administered immediately, and the advanced airway can be also placed early.

In our study, we demonstrated a higher odds ratio of survival to hospital discharge, ROSC, and neurological outcome in the early AAM group, but none were statistically significant. During the circulatory phase of CPR, early AAM might be beneficial for a favorable outcome [14] but controversy remains regarding the potentially beneficial effects of advanced airway management on neurological outcome.

Wong et al. [7] demonstrated that AAM before 5 minutes was associated with a better survival rate at hospital discharge (16.16 versus 11%). Better neurological outcomes were reported by Izawa et al. [9] (early AAM 2.2% versus late AAM 1.4%, aOR: 1.58 (95% CI; 1.24–2.02)) and Kajino et al. [15] with early prehospital AAM performed by EMS personnel (aOR for one minute delay, 0.91, 95% CI; 0.88 to 0.95).

A poor neurological outcome was reported by Wang et al. [10] in delayed AAM in hospitalized patients with a non-shockable rhythm (OR: 0.86, 95% CI; 0.80–0.93, *p* < 0.001). On the contrary, Hasegawa et al. [16] showed that a poor neurological outcome was more likely with AAM compared to conventional BMV (1.1% versus 2.9%, unadjusted OR: 0.38, 95% CI; 0.36 to 0.39). Andersen et al. [17] revealed that tracheal intubation during the first 15 minutes had lower rate of hospital discharge. According to the 2020 AHA guidelines for cardiopulmonary resuscitation and emergency cardiovascular care [4], there is no evidence that AAM is better than BMV.

Early AAM allows for high-quality CPR monitoring such as end-tidal carbon dioxide monitoring and asynchronous chest compression [18] and is associated with less gastric insufflation and aspiration. Moreover, it contributes oxygenation and ventilation control which may improve the outcome [5, 19]. In contrast to AAM, endotracheal tube intubation may interrupt chest compression, be poorly placed or inserted into the esophagus, and increase the risk of hyperventilation [20]. However, these adverse events would be diminished by an experienced physician and proper monitoring. Visualizing chest movements and chest auscultation, listening over epigastrium, and EtCO_2_ monitoring should be done immediately after intubation during CPR to ensure optimal CPR quality [12]. Training should be held frequently for physicians who perform intubation to improve tracheal intubation success rate, decrease interruption of chest compression, and reduce complications [4].

We emphasized the crucial role of high-quality CPR over advanced airway management. Interruption of chest compression should be minimized during resuscitation. Initiation of intubation should follow high-quality CPR.

### 4.1. Limitation

Our study had several limitations. First, the sample size was relatively small that reduced statistical power to detect small differences. We had to exclude a quarter of patients because of missing important data, which is the disadvantage of a retrospective study. Second, the rate and depth of chest compression were not recorded in the hospital notes nor the difficulty and success rate in achieving AAM. Finally, a supraglottic airway device was not used in any of our patients.

## 5. Conclusions

Our retrospective study did not demonstrate a significant improvement of survival to hospital discharge and survival with favorable neurological outcome in the patients with initial non-shockable cardiac arrest who underwent early AAM within two minutes. More research is needed on the timing of AAM and on airway management strategies to improve survival.

## Figures and Tables

**Table 1:** Study population characteristics.

Patient characteristics	All non-shockable rhythm cardiopulmonary arrest patients				
	Time to advanced airway management ≤2 min (*N* = 209)		Time to advanced airway management >2 min (*N* = 207)		*p* value
	*n*	%	n	%	
Gender					
(i) Male	125	49.60	127	50.40	0.764
(ii) Female	84	51.22	80	48.78	
Age (years) mean (SD)	62.32	(19.49)	60.62	(17.99)	0.358
Initial location of cardiac arrest					
(i) Out of hospital	166	50.46	163	49.54	0.904
(ii) In hospital	43	49.43	44	50.57	
Etiology of cardiac arrest					
(i) Presumed cardiac cause	126	53.85	108	46.15	0.216
(ii) Trauma	10	34.48	19	65.52	
(iii) Hypoxia	39	49.58	40	50.63	
(iv) Other medical causes	34	46.58	39	53.42	
Witnessed cardiac arrest	151	51.01	145	48.99	0.666
Bystander CPR	25	56.82	19	43.18	0.421
Collapsed time to first chest compression (minute) mean (SD)	21.34	(24.46)	25.97	(31.24)	0.09
Initial rhythm of cardiac arrest					
(i) Asystole	135	48.56	143	51.44	0.299
(ii) Pulseless electrical activity (PEA)	74	54.01	63	45.99	
Time to first dose of epinephrine (minute) mean (SD) group (minute)	1.39	(2.15)	2.28	(2.69)	<0.001
(i) 0–2	169	57.29	126	42.71	<0.001
(ii) Over 2	38	32.20	80	67.80	
Time to advanced airway management mean (SD)	1.16	(0.83)	5.87	(5.35)	—
Defibrillation during resuscitation	53	48.62	56	51.38	0.738
Intravenous amiodarone used during resuscitation	37	56.06	29	43.94	0.348
Intravenous calcium used during resuscitation	56	46.67	64	53.33	0.387
Intravenous sodium bicarbonate used during resuscitation	53	41.09	76	58.91	0.015

**Table 2:** Outcome of resuscitation between time to advanced airway management ≤2 minutes and >2 minutes.

Outcome	Time to advanced airway management ≤2 min (*N* = 209)		Time to advanced airway management >2 min (*N* = 207)		*p* value
	n	%	n	%	
Sustained ROSC	106/209	50.72	98/207	47.34	0.494
Survival to hospital discharge	23/209	11	14/207	6.80	0.168
Survival to hospital discharge with favorable neurological outcome	13/209	6.25	6/207	2.91	0.157

**Table 3:** Odds ratio from multivariate logistic regression predicting survival to hospital discharge from time to advance airway management ≤2 minutes VS >2 minutes.

Associated factor	Odds ratio	95% confidence interval	*p* value
Time to advanced airway management ≤2 min^*∗*^	1.28	0.59–2.76	0.524
Time to first dose of epinephrine within 2 min	1.63	0.60–4.74	0.317
Intravenous sodium bicarbonate used during resuscitation	0.21	0.06–0.74	0.015
Age	0.99	0.98–1.02	0.862
Gender	1.40	0.65–3.01	0.381
Etiology of cardiac arrest	0.93	0.68–1.28	0.652
Collapsed time to first chest compression	0.95	0.93–0.98	0.002

^*∗*^Reference with time to advanced airway management >2 minutes.

**Table 4:** Odds ratio from multivariable logistic regression predicting survival to hospital discharge with favorable neurological outcome from time to advance airway management ≤2 minutes and >2 minutes.

Associated factor	Odds ratio	95% confidence interval	*p* value
Time to advanced airway management ≤2 min^*∗*^	1.68	0.52–5.45	0.387
Time to first dose of epinephrine within 2 minutes	0.92	0.23–3.73	0.904
Intravenous sodium bicarbonate used during resuscitation	0.14	0.02–1.11	0.063
Age	0.99	0.96–1.02	0.536
Gender	1.74	0.57–5.28	0.328
Etiology of cardiac arrest	0.61	0.36–1.04	0.068
Collapsed time to first chest compression	0.31	0.86–0.97	0.003

^*∗*^Reference with time to advanced airway management >2 min.

## Data Availability

The data used to support the findings of this study are available from the corresponding author upon request.

## References

[1] Berdowski J., Berg R. A., Tijssen J. G. P., Koster R. W. (2010). Global incidences of out-of-hospital cardiac arrest and survival rates: systematic review of 67 prospective studies. *Resuscitation*.

[2] Bergum D., Nordseth T., Mjølstad O. C., Skogvoll E., Haugen B. O. (2015). Causes of in-hospital cardiac arrest-incidences and rate of recognition. *Resuscitation*.

[3] Srivilaithon W., Amnuaypattanapon K., Limjindaporn C. (2019). Predictors of in‐hospital cardiac arrest within 24 h after emergency department triage: a case-control study in urban Thailand. *Emergency Medicine Australasia*.

[4] Panchal A. R., Bartos J. A., Cabañas J. G. (2020). Part 3: adult basic and advanced life support: 2020 American heart association guidelines for cardiopulmonary resuscitation and emergency cardiovascular care. *Circulation*.

[5] Benoit J. L., Prince D. K., Wang H. E. (2015). Mechanisms linking advanced airway management and cardiac arrest outcomes. *Resuscitation*.

[6] Srivilaithon W. (2016). Prospective observational study of emergency airway management in emergency department. *Medical Journal of the Medical Association of Thailand*.

[7] Wong M. L., Carey S., Mader T. J., Wang H. E. (2010). Time to invasive airway placement and resuscitation outcomes after inhospital cardiopulmonary arrest. *Resuscitation*.

[8] Bobrow B. J., Clark L. L., Ewy G. A. (2008). Minimally interrupted cardiac resuscitation by emergency medical services for out-of-hospital cardiac arrest. *JAMA*.

[9] Izawa J., Iwami T., Gibo K. (2018). Timing of advanced airway management by emergency medical services personnel following out-of-hospital cardiac arrest: a population-based cohort study. *Resuscitation*.

[10] Wang C.-H., Chen W.-J., Chang W.-T. (2016). The association between timing of tracheal intubation and outcomes of adult in-hospital cardiac arrest: a retrospective cohort study. *Resuscitation*.

[11] Khera R., Chan P. S., Donnino M., Girotra S. (2016). Hospital variation in time to epinephrine for nonshockable in-hospital cardiac arrest. *Circulation*.

[12] Panchal A. R., Berg K. M., Hirsch K. G. (2019). American heart association focused update on advanced cardiovascular life support: use of advanced airways, vasopressors, and extracorporeal cardiopulmonary resuscitation during cardiac arrest: an update to the American heart association guidelines for cardiopulmonary resuscitation and emergency cardiovascular care. *Circulation*.

[13] Lupton J. R., Schmicker R., Daya M. R. (2019). Effect of initial airway strategy on time to epinephrine administration in patients with out-of-hospital cardiac arrest. *Resuscitation*.

[14] Weisfeldt M. L., Becker L. B. (2002). Resuscitation after cardiac arrest. *JAMA*.

[15] Kajino K., Iwami T., Kitamura T. (2011). Comparison of supraglottic airway versus endotracheal intubation for the pre-hospital treatment of out-of-hospital cardiac arrest. *Critical Care (London, England)*.

[16] Hasegawa K., Hiraide A., Chang Y., Brown D. F. M. (2013). Association of prehospital advanced airway management with neurologic outcome and survival in patients with out-of-hospital cardiac arrest. *JAMA*.

[17] Andersen L. W., Granfeldt A., Callaway C. W. (2017). Association between tracheal intubation during adult in-hospital cardiac arrest and survival. *JAMA*.

[18] Yeung J., Chilwan M., Field R., Davies R., Gao F., Perkins G. D. (2014). The impact of airway management on quality of cardiopulmonary resuscitation: an observational study in patients during cardiac arrest. *Resuscitation*.

[19] Spindelboeck W., Schindler O., Moser A. (2013). Increasing arterial oxygen partial pressure during cardiopulmonary resuscitation is associated with improved rates of hospital admission. *Resuscitation*.

[20] Aufderheide T. P., Sigurdsson G., Pirrallo R. G. (2004). Hyperventilation-induced hypotension during cardiopulmonary resuscitation. *Circulation*.

